# Physical and social neighborhood disorder in Latin American cities: a scoping review

**DOI:** 10.1590/0102-311XEN038423

**Published:** 2023-09-18

**Authors:** Amanda Silva Magalhães, Amanda Cristina de Souza Andrade, Bruno de Souza Moreira, Adalberto Aparecido dos Santos Lopes, Waleska Teixeira Caiaffa

**Affiliations:** 1 Observatório de Saúde Urbana de Belo Horizonte, Universidade Federal de Minas Gerais, Belo Horizonte, Brasil.; 2 Instituto de Saúde Coletiva, Universidade Federal de Mato Grosso, Cuiabá, Brasil.; 3 Núcleo de Estudos em Saúde Pública e Envelhecimento, Universidade Federal de Minas Gerais/Fundação Oswaldo Cruz, Belo Horizonte, Brasil.; 4 Grupo de Estudos e Pesquisa em Ambiente Urbano & Saúde, Universidade Federal de Santa Catarina, Florianópolis, Brasil.

**Keywords:** Residence Characteristics, Neighborhood Characteristics, Urban Health, Características de Residência, Características da Vizinhança, Saúde da População Urbana, Características de la Residencia, Características del Vecindario, Salud Urbana

## Abstract

Neighborhood disorder is an important aspect that may influence the health of residents in urban areas. The aims of this study were to map and systematize methods for measuring physical and social neighborhood disorder in studies conducted in Latin American cities. By means of a scoping review, articles published from 2000 in English, Spanish, and Portuguese with the following descriptors were mapped: neighborhood, physical disorder, and social disorder. Searches were conducted in MEDLINE (PubMed), LILACS (Virtual Health Library), Scopus, Web of Science, and Cochrane Library. Information on authorship, year, study type, locality, data source, target population, outcome, dominion, indicator, method, geographic unit, and unit of analysis was extracted. Variables from the disorder-related studies were extracted and grouped by similarity of content and themes. A total of 22 articles were identified, all published between 2012 and 2022, the majority in Brazil (n = 16). The perception of the individual was the most used method. The most frequent theme addressed in the physical disorder dominion was public streets (n = 20) and security (n = 15), in the social disorder dominion. A lack of consensus in the literature regarding variables used to measure physical and social neighborhood disorder in Latin American cities was detected. In addition to the need for standardization of the theme, studies to verify the sustainability of proposed measurement methods relevant to dynamically classify and compare urban neighborhoods and health impacts based on levels of exposure to physical and social disorder, are recommended.

## Resumo

A desordem da vizinhança é um importante aspecto que pode impactar a saúde de residentes em áreas urbanas. Os objetivos desta pesquisa foram mapear e sistematizar os métodos de mensuração da desordem física e social na vizinhança em estudos realizados em cidades da América Latina. Por meio de revisão de escopo, foram mapeados artigos publicados a partir do ano 2000 em inglês, espanhol e português com os seguintes descritores: vizinhança, desordem física e desordem social. As buscas foram realizadas no MEDLINE (PubMed), LILACS (Biblioteca Virtual em Saúde), Scopus, Web of Science e Biblioteca Cochrane. Foram extraídas informações sobre autoria, ano, tipo de estudo, local, fonte de dados, população-alvo, desfecho, domínio, indicador, método, unidade geográfica e unidade de análise. As variáveis dos estudos relacionadas à desordem foram extraídas e agrupadas pela similaridade dos conteúdos e temas. Foram identificados 22 artigos, publicados entre 2012 e 2022, sendo a maioria do Brasil (n = 16). A percepção do indivíduo foi o método mais utilizado. O tema mais frequentemente abordado no domínio da desordem física foi vias públicas (n = 20), enquanto no domínio social foi segurança (n = 15). Detectou-se ausência de consenso na literatura quanto às variáveis utilizadas para aferir a desordem física e social da vizinhança em cidades da América Latina. Além da necessidade de padronização do tema, recomendam-se estudos que verifiquem a sustentabilidade de métodos de mensuração propostos, relevantes para classificar e comparar, de forma dinâmica, vizinhanças urbanas e os impactos na saúde com base nos níveis de exposição à desordem física e social.

## Resumen

El desorden del vecindario es un aspecto importante que puede influir en la salud de los residentes en áreas urbanas. Los objetivos fueron mapear y sistematizar los métodos de medición del desorden físico y social en el vecindario en estudios realizados en ciudades de América Latina. Por medio de una revisión de alcance, fueron mapeados artículos publicados a partir del año 2000 en inglés, español y portugués, que poseían los siguientes descriptores: vecindario, desorden físico y desorden social. Las búsquedas se realizaron en MEDLINE (PubMed), LILACS (Biblioteca Virtual en Salud), Scopus, Web of Science y Librería Cochrane. Se extrajeron informaciones sobre la autoría, el año, el tipo de estudio, la ubicación, la fuente de datos, la población objetivo, el resultado, el dominio, el indicador, el método, la unidad geográfica y la unidad de análisis. Las variables de los estudios relacionados con el desorden fueron extraídas y agrupadas por la similitud de los contenidos y temas. Fueron identificados 22 artículos, todos publicados entre 2012 y 2022, siendo la mayoría de Brasil (n = 16). La percepción del individuo fue el método más utilizado. El tema más frecuente abordado en el ámbito del desorden físico fue el de las vías públicas (n = 20) y seguridad (n = 15), en lo social. Se detectó una falta de consenso en la literatura en cuanto a las variables utilizadas para medir el desorden físico y social del vecindario en ciudades de América Latina. Además de la necesidad de estandarización del tema, se recomiendan estudios que verifiquen la sostenibilidad de los métodos de medición propuestos, relevantes para clasificar y comparar, de forma dinámica, los vecindarios urbanos y los impactos en la salud con base en niveles de exposición a desorden físico y social.

## Introduction

Urbanization is a global trend that is characterized as a dynamic process with differentiated patterns in each region of the world. Currently, 55% of the world population resides in urban areas. It is estimated that this figure will increase to 68% by 2050, with most of the growth occurring in low- and middle-income countries ^1^.

Considered the most urbanized region in the world, Latin America has about 80% of residents in urban areas, which is a higher proportion than high-income countries ^2^. This accelerated urbanization process has resulted in insufficient infrastructure, environmental deterioration, the formation of subnormal settlements and, most importantly, the region present the greatest socioeconomic inequality in the world ^3^.

Recent studies have focused on specifically investigating the context of neighborhoods, since individual characteristics alone are insufficient and fail to capture important determinants of health ^4^ ^,^ ^5^ ^,^ ^6^. The physical and social characteristics of neighborhoods can influence health by the availability and accessibility of health services, infrastructure, and green spaces, among others ^7^.

Among neighborhood characteristics, the concept of disorder stands out. It is related to social and structural disorganization, may influence social control and increase the levels of violence, crime and other negative outcomes ^8^ ^,^ ^9^ ^,^ ^10^. Studies describe disorder as visible signs of neglect and degradation, indicating a disruption of order and social control that can consequently impair individuals’ quality of life ^8^ ^,^ ^11^ ^,^ ^12^ ^,^ ^13^.

Disorder is classified by some authors into two components. The physical, which is related to the characteristics of a certain spatial context, and the social, which directly involves people ^14^. Physical disorder can be exemplified by empty, abandoned, vandalized and degraded buildings, abandoned cars, graffiti, noise, and garbage in the streets. Social disorder includes certain types of behavior in public places, such as people under the influence of drugs or alcohol, drug dealing, hostile discussions, conflicts and fights, the presence of lazy and criminal people and gang activity, prostitution, and high levels of police activity ^8^ ^,^ ^14^.

Studies in Latin America show that disorder can impact different health outcomes, such as walking ^15^, perception of insecurity and fear of crime ^16^, use of parks ^17^, self-perception of health ^13^ ^,^ ^18^ ^,^ ^19^ ^,^ ^20^, satisfaction with life ^21^, and occurrence of homicides ^22^. As the literature on the subject increases, there is also a growth in the availability of measurement methods ^23^.

Recent studies have reviewed methods for assessing the physical and social attributes of context. Among them are two systematic reviews ^23^ ^,^ ^24^, two literature reviews ^25^ ^,^ ^26^, and a scoping review ^27^. However, these reviews selected English-language articles, and one of them also included Dutch-language articles, which may have resulted in a selection bias, with fewer studies produced in Latin America. Furthermore, these studies did not aim to specifically assess neighborhood disorder. An exception is the one by Ndjila et al. ^11^ which presented a brief literature review providing a summary of data collection methods, terms, and specific items employed to assess neighborhood order and disorder. However, only English language studies were included in it and again, the Latin American context was not considered.

Although scoping reviews are less used when aiming to evaluate the quality of the evidence presented ^28^, it is considered an adequate approach to study the main concepts that sustain a field of research, notably when regarding constructs under development, which need standardized empirical support. Thus, the objective of this study was to map and systematize the methods for measuring neighborhood disorders in studies conducted in Latin American cities, through a scoping review.

## Methods

### Protocol and registration

This scoping review was developed based on the recommendations of the international guide *Preferred Reporting Items for Systematic reviews and Meta-Analyses extension for Scoping Reviews* (PRISMA-ScR) ^29^ and by the method proposed by the Joanna Briggs Institute ^28^. The protocol was registered in the Open Science Framework (https://osf.io/8rj4y) on December 30, 2021.

To orient and address the development of specific inclusion criteria for this review, the following guiding question was formulated by the Population, Concept and Context (PCC) strategy ^28^: “What are the concepts and methods used to measure neighborhood disorder in Latin American cities?” Thus, the following were defined: Population - neighborhoods; Concept - methods in urban health to measure physical and social disorder; and Context - geographic units of cities in Latin America.

### Eligibility criteria

We included in the revision articles that had full-text availability published from the year 2000 in English, Spanish, and Portuguese, and that contained anywhere in the text the descriptor “neighborhood” and the keywords “physical disorder” or “social disorder”.

Articles that did not measure neighborhood disorder in Latin American cities, as well as reviews, editorials, essays, and opinion articles were excluded.

### Information sources

Searches were conducted in December 2022 in MEDLINE (via PubMed), LILACS (via Virtual Health Library), Scopus (via CAPES Portal), Web of Science (via CAPES Portal), and Cochrane Library (via CAPES Portal) databases.

The references of the selected articles were checked to find additional studies not identified in the previous searches, considering the previously established eligibility criteria.

### Search strategy

The search strategy was developed considering the inclusion criteria for the MEDLINE database (via PubMed), using the Health Science Descriptors (DeCS) and keywords: *(“neighborhood”) AND (“physical disorder” OR “social disorder”)* (Supplementary Material: https://cadernos.ensp.fiocruz.br/static//arquivo/suppl-en-00038423_3467.pdf).

This search strategy was adapted according to the specificities of each database used. In all of them, the search was performed considering December 15, 2022 as the publication deadline.

The final search results were exported to Mendeley (https://www.mendeley.com), so that duplicate articles were removed.

### Selecting sources of evidence

Article titles and abstracts were initially examined by one reviewer. The articles then selected were read in full by two independent reviewers, who identified whether the articles met the inclusion criteria. Disagreements were discussed with a third reviewer to reach consensus.

### Data collection process

A structured instrument was designed to extract and synthesize the main elements found in each selected article, and Microsoft Excel (https://products.office.com/) was used for data tabulation.

### Extracted information

Data extracted included authorship, year of publication, type of study (psychometric analysis; application of the method; association), locality, data source (on-site audit; secondary data; interview), target population (adolescents; youths; adults; older adults). Moreover, information such as outcome (when applicable), dominion of disorder (physical; social), type of indicator (simple; composite), method (demographic census; systematic social observation (SSO); perception of the individual; other), geographic unit of data collection of the disorder variables (street segment; census tract; neighborhood) and unit of analysis of disorder (individual; context), were systematized after reading the full articles (Box 1). The original variables of the physical and social disorder indicator were distributed into categories (Box 2).

**Box 1:** Key characteristics of the studies included in the scoping review (n = 22).

STUDY	TIPE OF STUDY	LOCALITY	DATA SOURCE	TARGET POPULATION	OUTCOME
Moran et al. ^31^ (2022)	Association	Buenos Aires (Argentina), Bogotá (Colombia), Caracas (Venezuela), Fortaleza (Brazil), La Paz (Bolivia), Lima (Peru), Mexico City (Mexico), Montevideo (Uruguay), Panama City (Panama), Quito (Ecuador), and São Paulo (Brazil)	Interview	Adults	Social disorder
Moreira et al. ^42^ (2022)	Association	Belo Horizonte (Brazil)	Interview	Older adults	Walking for transportation
Moreira et al. ^15^ (2021)	Association	Brazil	Interview	Older adults	Global walk
Auler et al. ^12^ (2020)	Association	Brazil	Secondary data	Adolescents	Common mental disorders
Layera et al. ^16^ (2020)	Association	Santiago (Chile)	Secondary data	Adults	Feeling of insecurity and fear of crime
Moran et al. ^17^ (2020)	Association	Buenos Aires (Argentina), Bogotá (Colombia), Caracas (Venezuela), Fortaleza (Brazil), La Paz (Bolivia), Lima (Peru), Mexico City (Mexico), Montevideo (Uruguay), Panama City (Panama), Quito (Ecuador), and São Paulo (Brazil)	Interview	Adults	Regular use of parks, squares or green areas
Moreira et al. ^40^ (2020)	Association	Belo Horizonte (Brazil)	Interview	Older adults	History of falls
Vaz et al. ^13^ (2020)	Association	Buenos Aires (Argentina), Mexico City (Mexico), Panama City (Panama), and Lima (Peru)	Interview	Adults	Self-rated health
Andrade et al. ^44^ (2019)	Association	Belo Horizonte (Brazil)	On-site audit	Adults	Leisure physical activity
Parajára et al. ^43^ (2019)	Association	Vespasiano (Brazil)	Interview	Adolescents	Screen time
Remigio et al. ^46^ (2019)	Application of the method *	Rio de Janeiro (Brazil)	On-site audit	NA	NA
Vaz et al. ^21^ (2019)	Association	Belo Horizonte (Brazil)	On-site audit	Older adults	Satisfaction with life
Vilalta et al. ^22^ (2019)	Association	Mexico City (Mexico)	Secondary data	Youths	Occurrence of homicide
Zanelatto et al. ^41^ (2019)	Association	Florianopolis (Brazil)	Interview	Adults	Blood pressure levels
Costa et al. ^45^ (2017)	Psychometric analysis **	Belo Horizonte (Brazil)	On-site audit	NA	NA
Höfelmann et al. ^18^ (2015)	Association	Florianopolis (Brazil)	Interview	Adults	Self-rated health
Meireles et al. ^20^ (2015)	Association	Belo Horizonte (Brazil)	Interview	Adults	Self-rated health
Rodrigues et al. ^19^ (2015)	Association	Belo Horizonte (Brazil)	Interview	Adults	Self-rated health
Célio et al. ^38^ (2014)	Association	Belo Horizonte (Brazil)	Interview	Adults	Self-perceived neighborhood extension
Höfelmann et al. ^30^ (2013)	Psychometric analysis **	Florianopolis (Brazil)	Interview	Adults	NA
Friche et al. ^39^ (2013)	Psychometric analysis **	Belo Horizonte (Brazil)	Interview	Adults	NA
Escobar ^59^ (2012)	Association	Bogotá (Colombia)	Secondary data	Youths	Homicides rate

NA: not applicable.

* The study adapted the on-site audit strategies using smartphones to apply systematic social observation;

** Construct validation studies and internal consistency analysis;

*** The outcome of the study was the cumulative homicide rate calculated from the sum of the homicides in the years 2003, 2004, and 2005 divided by the average population size in the three years and then multiplied by 10,000.

**Box 2:** Classification of the original neighborhood disorder variables according to domain, theme and category.

DOMAIN	THEME	CATEGORY	ORIGINAL VARIABLES	STUDY
Physical disorder	Environmental factors	Music	Places with loud music	^38^ ^,^ ^39^ ^,^ ^40^ ^,^ ^42^
Physical disorder	Environmental factors	Music	Loud music	^19^
Physical disorder	Environmental factors	Odor	Unpleasant odor	^18^ ^,^ ^30^ ^,^ ^46^
Physical disorder	Environmental factors	Air, water and ground pollution	Air, water and ground pollution	^18^ ^,^ ^30^
Physical disorder	Aesthetics	Trees	Trees	^12^ ^,^ ^31^
Physical disorder	Aesthetics	Pleasant places	No safe place for children to play	^18^ ^,^ ^30^
Physical disorder	Aesthetics	Pleasant places	Pleasant neighborhood for children, young people and teenagers	^19^ ^,^ ^38^ ^,^ ^39^ ^,^ ^40^ ^,^ ^42^
Physical disorder	Real estate and public facilities	Deteriorated properties; Graffiti on buildings and public facilities	Properties with graffiti and signs of deterioration	^45^
Physical disorder	Real estate and public facilities	Deteriorated properties; Graffiti on buildings and public facilities; Empty properties, lots and lands	Buildings and houses with graffiti, broken windows, damaged walls, or abandoned	^15^
Physical disorder	Real estate and public facilities	Deteriorated properties	Buildings or houses in poor condition	^16^
Physical disorder	Real estate and public facilities	Graffiti on buildings and public facilities	Graffiti on walls	^16^
Physical disorder	Real estate and public facilities	Graffiti on buildings and public facilities	Graffiti	^22^
Physical disorder	Real estate and public facilities	Graffiti on buildings and public facilities	Graffiti on public equipment and facilities	^21^
Physical disorder	Real estate and public facilities	Graffiti on buildings and public facilities	Graffiti on public equipments	^44^ ^,^ ^45^
Physical disorder	Real estate and public facilities	Buildings under construction	Buildings under construction	^19^ ^,^ ^31^
Physical disorder	Real estate and public facilities	Empty properties, lots and lands	Empty lots and lands	^19^
Physical disorder	Real estate and public facilities	Empty properties, lots and lands	Abandoned, illegally taken or invaded buildings, houses or lots	^13^
Physical disorder	Real estate and public facilities	Empty properties, lots and lands	Empty buildings, houses or lots	^38^ ^,^ ^39^ ^,^ ^40^ ^,^ ^42^
Physical disorder	Neighborhood problems	Points of sale and use of drugs	Buying and using drugs	^13^
Physical disorder	Neighborhood problems	Vandalism	People breaking windows, damaging walls, or committing vandalism	^38^ ^,^ ^39^ ^,^ ^40^ ^,^ ^42^
Physical disorder	Public streets	Holes	Holes in the streets	^16^
Physical disorder	Public streets	Sidewalks	Sidewalks	^12^
Physical disorder	Public streets	Sidewalks	Uneven sidewalks	^18^ ^,^ ^30^
Physical disorder	Public streets	Sidewalks	Uneven pavement	^41^
Physical disorder	Public streets	Open sewer	Open sewer	^12^
Physical disorder	Public streets	Displaced electrical wires (hanging, tangled, or knocked down)	Downed electrical wires	^22^
Physical disorder	Public streets	Displaced electrical wires (hanging, tangled, or knocked down)	Dangling or tangled electrical wires	^46^
Physical disorder	Public streets	Street lighting	Street lighting	^12^ ^,^ ^19^ ^,^ ^38^ ^,^ ^39^ ^,^ ^40^ ^,^ ^42^
Physical disorder	Public streets	Street lighting	Poorly lit streets	^13^ ^,^ ^31^
Physical disorder	Public streets	Garbage	Garbage dumps	^13^
Physical disorder	Public streets	Garbage	Garbage	^12^ ^,^ ^18^ ^,^ ^19^ ^,^ ^30^ ^,^ ^31^ ^,^ ^46^
Physical disorder	Public streets	Garbage	Garbage (needles, cigarettes, cans and condoms)	^21^ ^,^ ^44^ ^,^ ^45^
Physical disorder	Public streets	Garbage; Bushes	Garbage or tall grass	^15^ ^,^ ^38^ ^,^ ^39^ ^,^ ^40^ ^,^ ^42^
Physical disorder	Public streets	Garbage	Presence of garbage	^41^
Physical disorder	Public streets	Curbs	Curbs	^12^
Physical disorder	Public streets	Curbs	Curb ramp	^12^
Physical disorder	Public streets	Paving	Paving	^12^
Physical disorder	Public streets	Rats or signs of them	Rats or signs of them in the street	^15^
Physical disorder	Public streets	Street gutters	Street gutters	^12^
Physical disorder	Public streets	Public transportation	Absence of public transport	^18^ ^,^ ^30^
Physical disorder	Public streets	Public transportation	Urban transport	^41^
Physical disorder	Public streets	Leakage of water, gas and sewer	Drinking water leak	^22^
Physical disorder	Public streets	Leakage of water, gas and sewer	Sewer leak	^22^
Physical disorder	Public streets	Leakage of water, gas and sewer	Gas leak	^22^
Physical disorder	Public streets	Abandoned vehicles	Abandoned vehicles	^22^
Physical disorder	Public streets	Traffic speed	High speed cars	^18^ ^,^ ^30^
Physical disorder	Public streets	Traffic speed	Traffic speed	^41^
Social disorder	Environmental factors	Music	Loud music	^43^
Social disorder	Environmental factors	Music	Public disturbance in the form of a party	^22^
Social disorder	Environmental factors	Music	People or places in the neighborhood where loud music is heard	^20^
Social disorder	Environmental factors	Odor	Unpleasant odor caused by neighbors	^16^
Social disorder	Environmental factors	Noises	Public disturbance in form of street scandal	^22^
Social disorder	Environmental factors	Noises	Nuisance noises	^16^
Social disorder	Real estate and public facilities	Video game, lottery and gambling establishments	Video game, lottery and gambling establishments	^59^
Social disorder	Real estate and public facilities	Empty properties, lots and lands	Abandoned buildings, houses, or warehouses with broken windows or doors	^43^
Social disorder	Real estate and public facilities	Empty properties, lots and lands	Abandoned buildings, houses or sheds	^20^
Social disorder	Neighborhood problems	Indecent assault	Public indecency	^22^
Social disorder	Neighborhood problems	Indecent assault	Record of person urinating in public	^22^
Social disorder	Neighborhood problems	Bad reputation reported from the neighborhood	Bad neighborhood reputation	^18^ ^,^ ^30^ ^,^ ^41^
Social disorder	Neighborhood problems	Begging	Indigence or begging	^13^ ^,^ ^17^
Social disorder	Neighborhood problems	Points of sale and consumption of alcohol	Alcoholic beverage establishments	^59^
Social disorder	Neighborhood problems	Points of sale and use of drugs	People using or selling illegal drugs	^19^ ^,^ ^31^ ^,^ ^38^ ^,^ ^39^ ^,^ ^40^ ^,^ ^42^
Social disorder	Neighborhood problems	Points of sale and use of drugs	Use of drugs	^17^ ^,^ ^18^ ^,^ ^30^ ^,^ ^41^
Social disorder	Neighborhood problems	Points of sale and use of drugs	Public use of drugs and alcohol	^22^
Social disorder	Neighborhood problems	Prostitution	Prostitution	^13^ ^,^ ^17^ ^,^ ^19^ ^,^ ^31^ ^,^ ^38^ ^,^ ^39^ ^,^ ^40^ ^,^ ^42^
Social disorder	Neighborhood problems	Vandalism	Vandalism	^18^ ^,^ ^30^ ^,^ ^41^
Social disorder	Security	Assaults, arguments and offenses	Aggressive acts or offenses	^13^
Social disorder	Security; Environmental factors	Assaults, arguments and offenses; Music	People arguing loudly or having parties late into the night	^20^
Social disorder	Security	Assaults, murders and kidnappings	Assault or crime	^17^ ^,^ ^31^
Social disorder	Security	Assaults, murders and kidnappings	Assaults	^18^ ^,^ ^30^ ^,^ ^41^
Social disorder	Security	Assaults, murders and kidnappings	Murders	^18^ ^,^ ^30^ ^,^ ^41^
Social disorder	Security	Assaults, murders and kidnappings	Robberies	^41^
Social disorder	Security	Assaults, murders and kidnappings	Kidnappings	^41^
Social disorder	Security	Noise of gunfire	Noise of gunfire	^20^
Social disorder	Security	Walk after dark	Walking in the neighborhood after dark	^41^
Social disorder	Security	Walk after dark	Insecurity when walking after dark	^18^ ^,^ ^30^
Social disorder	Security	Conflicts between neighbors	Conflicts between neighbors	^13^
Social disorder	Security	Criminals in the neighborhood	Robbers	^18^ ^,^ ^30^
Social disorder	Security	Criminals in the neighborhood	Gangs activities	^13^
Social disorder	Security	Criminals in the neighborhood	Criminals walking around the neighborhood	^38^ ^,^ ^39^ ^,^ ^40^ ^,^ ^42^
Social disorder	Security	Criminals in the neighborhood	Criminals or thieves walking in the neighborhood	^19^
Social disorder	Security	Criminals in the neighborhood	Gangs	^17^ ^,^ ^31^
Social disorder	Security	Criminals in the neighborhood	People who carry guns (other than police)	^20^
Social disorder	Security	Problems with the police	Problems with the police	^18^ ^,^ ^30^ ^,^ ^41^
Social disorder	Security	Risk of violence	Person at risk registration	^22^
Social disorder	Public streets	Garbage	Garbage	^22^
Social disorder	Public streets	Garbage	Trash or rubble	^20^ ^,^ ^43^
Social disorder	Public streets	Garbage; Bushes	Vacant lots filled with rubbish and rubble or overgrown with bushes	^20^
Social disorder	Public streets	Garbage	Presence of garbage in the streets	^16^

### Summary of results

First, for each article included in this review, the dominion of disorder in the neighborhood, physical and/or social, defined according to each author, was identified. Only the studies by Höfelmann et al. ^18^ ^,^ ^30^ named the physical disorder dominion as physical neighborhood problems.

Next, the original variables used in the articles to measure disorder were extracted and grouped into categories by content similarity. For example, the variable “poorly lit streets” ^13^ ^,^ ^31^ was included in the category street lighting and “unsafe walking after dark in the neighborhood” ^18^ ^,^ ^30^ in the category walking after dark.

A total of 95 original variables were identified, 51 being physical disorder and 44 social disorder, which in turn were grouped into 41 categories. Finally, the variables were regrouped into themes: environmental factors, aesthetics, real estate and public facilities, neighborhood problems, security, and public streets. The environmental factors theme includes variables related to noise, odor, and pollution. Aesthetics includes variables that indicate whether a place is pleasant and the presence of trees. The real estate and public facilities theme includes items that characterize types of buildings, graffiti on buildings and public facilities. The neighborhood problems theme includes variables that are considered nuisances experienced by the residents, such as the presence of points of drug sales and consumers of alcohol and drugs. The security theme includes violent situations and presence of criminals. And in the public streets theme are the items that characterize problems in streets and sidewalks, such as holes, lack of public lighting, garbage, and others.

The themes that presented the highest number of categories were public streets (n = 15 categories) for physical disorder, and security (n = 8 categories) and neighborhood problems (n = 7 categories) for social disorder (Boxes 2 and 3).

**Box 3:** Disorder-related characteristics of the studies included in the scoping review (n = 22).

STUDY	DOMAIN	INDICATOR *	METHOD	GEOGRAPHIC UNIT OF DATA COLLECTION OF DISORDER	UNIT OF ANALYSIS OF DISORDER
Moran et al. ^31^ (2022)	Physical and social disorder	Composite	Perception of the individual	Neighborhood	Individual
Moreira et al. ^42^ (2022)	Physical and social disorder	Composite	Perception of the individual	Neighborhood	Individual
Moreira et al. ^15^ (2021)	Physical disorder	Simple	Perception of the individual	Neighborhood	Individual
Auler et al. ^12^ (2020)	Physical disorder	Composite	Demographic census **	Census tract	Context ***
Layera et al. ^16^ (2020)	Physical and social disorder	Composite	Demographic census, satellite images, and administrative data	Census tract	Context ***
Moran et al. ^17^ (2020)	Social disorder	Simple	Perception of the individual	Neighborhood	Individual
Moreira et al. ^40^ (2020)	Physical and social disorder	Composite	Perception of the individual	Neighborhood	Context ***
Vaz et al. ^13^ (2020)	Physical disorder	Composite	Systematic social observation	Neighborhood	Context ***
Andrade et al. ^44^ (2019)	Physical disorder	Composite	Perception of the individual	Street segment	Context ***
Parajára et al. ^43^ (2019)	Social disorder	Simple	Systematic social observation	Neighborhood	Individual
Remigio et al. ^46^ (2019)	Physical disorder	Simple	Systematic social observation	Street segment	Context ***
Vaz et al. ^21^ (2019)	Physical disorder	Composite	Demographic census, satellite images, and administrative data	Street segment	Context ***
Vilalta et al. ^22^ (2019)	Physical and social disorder	Composite	Crowding source ^#^	Neighborhood	Context ***
Zanelatto et al. ^41^ (2019)	Physical and social disorder	Composite	Perception of the individual	Census tract	Context ***
Costa et al. ^45^ (2017)	Physical disorder	Composite	Systematic social observation	Street segment	Context ***
Höfelmann et al. ^18^ (2015)	Physical ^##^ and social disorder	Composite	Perception of the individual	Neighborhood	Context ***
Meireles et al. ^20^ (2015)	Social disorder	Simple	Perception of the individual	Neighborhood	Individual
Rodrigues et al. ^19^ (2015)	Physical and social disorder	Composite	Perception of the individual	Neighborhood	Individual
Célio et al. ^38^ (2014)	Physical and social disorder	Composite	Perception of the individual	Neighborhood	Individual
Höfelmann et al. ^30^ (2013)	Physical ^##^ and social disorder	Composite	Perception of the individual	Neighborhood	Context ***
Friche et al. ^39^ (2013)	Physical and social disorder	Composite	Perception of the individual	Neighborhood	Context ***
Escobar ^59^ (2012)	Social disorder ^###^	Composite	Demographic census	Census tract	Context ***

* The simple indicator shows that the disorder variables were assessed separately, while the composite indicator shows that the disorder variables were grouped and presented in scales;

** Collection of the surrounding characteristics for this study was conducted by the 2010 *Demographic Census* supervisors;

*** Aggregated into a geographic unit (e.g., census tract, municipality);

^#^ The source of mass collaboration information used in this study was Mexico City’s 911 calling system, which receives emergency and non-emergency requests by phone and message, allowing direct request for services or crime reports;

^##^ The studies used the term physical problems to refer to physical disorder;

^###^ The study used two indicators as a proxy for disorder obtained from the 2005 *Demographic Census*.

## Results

**Figure 1:**
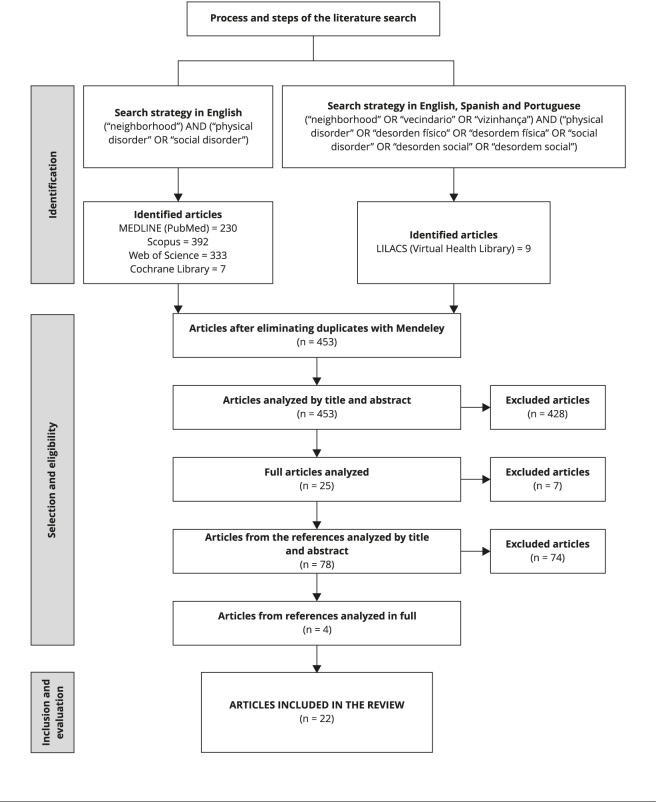
Flowchart for article selection.

The search strategy identified 971 articles, of which 518 were excluded as they were duplicates. The titles and abstracts of the remaining 453 articles were read, and 428 were excluded for not meeting the inclusion criteria. Then, the 25 articles were read in full, and out of these, seven were excluded for not describing the disorder indicator (n = 6) and for not having as an objective to evaluate the disorder (n = 1). After checking the references of the selected articles, additional 78 articles were evaluated using the title and abstract. Out of these 74 articles were excluded for not meeting the inclusion criteria. Then, the remaining four articles were read in full, and all were included. In the end, 22 articles comprised the present review (Figure 1).

Of the 22 articles included, 18 were association studies, three were psychometric analyses, and only one was an application of the method. In Brazil, the articles were developed in Belo Horizonte (Minas Gerais State) (n = 9), Florianópolis (Santa Catarina State) (n = 3), Rio de Janeiro (n = 1), Vespasiano (Minas Gerais State) (n = 1) and set of cities representative of the country (n = 2). The remainder was conducted in Bogotá, Colombia (n = 1), Mexico City, Mexico (n = 1), Santiago, Chile (n = 1) and other Latin American cities (n = 3). The publication period was from 2012 to 2022 (Box 1).

The data collection sources were: interview (n = 14), on-site audit (n = 4) and secondary data (n = 4). As for target population, the studies were conducted with adults (n = 12), older adults (n = 4), youths (n = 2) and adolescents (n = 2). The health outcomes of the included studies were quite varied, with self-rated health being the most frequent (n = 4) (Box 1).

Among the included articles, six assessed only the physical disorder dominion, four only the social disorder, and 12 assessed both physical and social disorder dominions. Regarding the disorder indicator, most articles presented composite indicators (n = 17) (Box 3).

The main methods used to measure disorder were individual perception (n = 14), SSO (n = 4), secondary data from each country’s demographic census information (n = 3) and one of the articles also used satellite images and administrative data. In addition, one of the studies employed a crowding source of information (Box 3).

The geographic units of data collection for the disorder variables were neighborhood (n = 14), census tract (n = 4) and street segments (n = 4); and the units of analysis for physical and social disorder were context (n = 14) and individual (n = 8) (Box 3).

Of the six themes defined, four were present in both dominions of disorder: environmental factors, real estate and public facilities, neighborhood problems, and public streets. The aesthetic theme (n = 7) was observed only for physical disorder and the security theme (n = 14) for social disorder. Public streets (n = 20) and real estate and public facilities (n = 12) were the most frequent in the physical disorder dominion, and security (n = 15) and neighborhood problems (n = 13) in the social disorder dominion (Box 4).

**Box 4:** Relationship of studies according to theme, category and type of disorder.

THEME/CATEGORY	PHYSICAL DISORDER	SOCIAL DISORDER
ENVIRONMENTAL FACTORS (n)	3	3
Music	^19^ ^,^ ^38^ ^,^ ^39^ ^,^ ^40^ ^,^ ^42^	^20^ ^,^ ^22^ ^,^ ^43^
Odor	^18^ ^,^ ^30^	^16^ ^,^ ^46^
Air, water and ground pollution	^18^ ^,^ ^30^	^-^
Noises	^-^	^16^ ^,^ ^62^
AESTHETICS (n)	2	0
Trees	^12^ ^,^ ^31^	^-^
Pleasant places	^19^ ^,^ ^38^ ^,^ ^39^ ^,^ ^40^ ^,^ ^42^	^-^
REAL ESTATE AND PUBLIC FACILITIES (n)	4	2
Video game, lottery and gambling establishments	^-^	^59^
Deteriorated properties	^15^ ^,^ ^16^ ^,^ ^40^ ^,^ ^45^	^-^
Graffiti on buildings and public facilities	^15^ ^,^ ^16^ ^,^ ^21^ ^,^ ^44^ ^,^ ^45^	^-^
Buildings under construction	^19^ ^,^ ^31^ ^,^ ^38^ ^,^ ^39^	^-^
Empty properties, lots and lands	^13^ ^,^ ^15^ ^,^ ^19^ ^,^ ^38^ ^,^ ^39^ ^,^ ^40^ ^,^ ^42^	^20^ ^,^ ^43^
NEIGHBORHOOD PROBLEMS (n)	2	7
Indecent assault	^-^	^22^
Bad reputation reported from the neighborhood	^-^	^18^ ^,^ ^30^ ^,^ ^41^
Begging	^-^	^13^ ^,^ ^17^
Points of sale and consumption of alcohol	^-^	^22^ ^,^ ^59^
Points of sale and use of drugs	^13^	^18^ ^,^ ^19^ ^,^ ^22^ ^,^ ^30^ ^,^ ^31^ ^,^ ^38^ ^,^ ^39^ ^,^ ^40^ ^,^ ^41^ ^,^ ^42^
Prostitution	^-^	^13^ ^,^ ^17^ ^,^ ^19^ ^,^ ^31^ ^,^ ^38^ ^,^ ^39^ ^,^ ^40^ ^,^ ^42^
Vandalism	^31^ ^,^ ^38^ ^,^ ^39^ ^,^ ^40^ ^,^ ^42^	^18^ ^,^ ^30^ ^,^ ^41^
SECURITY (n)	0	8
Assaults, arguments and offenses	^-^	^13^ ^,^ ^20^ ^,^ ^22^ ^,^ ^62^
Assaults, murders and kidnappings	^-^	^17^ ^,^ ^18^ ^,^ ^30^ ^,^ ^31^ ^,^ ^41^
Noise of gunfire	^-^	^20^
Walk after dark	^-^	^18^ ^,^ ^30^ ^,^ ^41^
Conflicts between neighbors	^-^	^13^
Criminals in the neighborhood	^-^	^18^ ^,^ ^19^ ^,^ ^20^ ^,^ ^31^ ^,^ ^38^ ^,^ ^39^ ^,^ ^40^ ^,^ ^42^
Problems with the police	^-^	^18^ ^,^ ^30^ ^,^ ^41^
Risk of violence	^-^	^22^
PUBLICS STREETS (n)	15	2
Holes	^16^	^-^
Sidewalks	^12^ ^,^ ^17^ ^,^ ^18^ ^,^ ^30^ ^,^ ^41^	^-^
Open sewer	^12^	^-^
Displaced electrical wires (hanging, tangled, or knocked down)	^22^ ^,^ ^46^	^-^
Street lighting	^12^ ^,^ ^13^ ^,^ ^15^ ^,^ ^17^ ^,^ ^19^ ^,^ ^31^ ^,^ ^38^ ^,^ ^39^ ^,^ ^40^ ^,^ ^42^	^-^
Garbage	^12^ ^,^ ^13^ ^,^ ^15^ ^,^ ^18^ ^,^ ^19^ ^,^ ^21^ ^,^ ^30^ ^,^ ^31^ ^,^ ^38^ ^,^ ^39^ ^,^ ^40^ ^,^ ^41^ ^,^ ^42^ ^,^ ^44^ ^,^ ^45^ ^,^ ^46^	^16,20,22,43^
Bushes	^15^ ^,^ ^40^	^20^
Curbs	^12^	^-^
Paving	^12^ ^,^ ^17^	^-^
Rats or signs of them	^15^	^-^
Street gutters	^12^	^-^
Public transportation	^18^ ^,^ ^30^ ^,^ ^41^	^-^
Leakage of water, gas and sewer	^22^	^-^
Abandoned vehicles	^22^ ^,^ ^62^	^-^
Traffic speed	^18^ ^,^ ^30^ ^,^ ^41^	^-^

## Discussion

This scoping review identified 22 articles published between 2012 and 2022 that assessed physical and social disorder in Latin American cities. Most studies were conducted in Brazil and used the perception of the individual to measure disorder. Public streets and real estate and public facilities were the most frequent themes to measure physical disorder, while for social disorder the themes were security and neighborhood problems.

As the number of studies evaluating disorders increased, there was also an increase in the choice of variables used to describe them ^11^. Some studies suggested that physical disorder and social disorder might overlap ^32^ ^,^ ^33^. However, most authors advocated a distinction between these components ^34^ ^,^ ^35^ ^,^ ^36^ ^,^ ^37^. For example, drug outlets and drug use was reported in one of the included studies as physical disorder ^13^. However, due to its behavioral nature, it was more described as social disorder ^18^ ^,^ ^19^ ^,^ ^22^ ^,^ ^30^ ^,^ ^31^ ^,^ ^38^ ^,^ ^39^ ^,^ ^40^ ^,^ ^41^ ^,^ ^42^. Similarly, the presence of garbage, which was reported as social disorder in some studies ^16^ ^,^ ^20^ ^,^ ^22^ ^,^ ^43^, was more often considered as physical disorder because it is a characteristic of the physical environment ^12^ ^,^ ^13^ ^,^ ^15^ ^,^ ^18^ ^,^ ^19^ ^,^ ^21^ ^,^ ^30^ ^,^ ^31^ ^,^ ^38^ ^,^ ^39^ ^,^ ^40^ ^,^ ^41^ ^,^ ^42^ ^,^ ^44^ ^,^ ^45^ ^,^ ^46^. We also observed that the presence of abandoned or deteriorated property and equipment was used to measure social disorder ^20^ ^,^ ^43^, whereas in most of the studies evaluated, it was considered as physical disorder ^13^ ^,^ ^15^ ^,^ ^16^ ^,^ ^19^ ^,^ ^38^ ^,^ ^39^ ^,^ ^40^ ^,^ ^42^ ^,^ ^45^. Therefore, it was found that there is no consensus in the literature about the distribution of the variables for the evaluation of physical and social disorder in Latin America, which becomes a challenge for the systematization of research and comparison among studies.

Importantly, most of the studies evaluated used the individual’s perception to obtain information of the disorder ^13^ ^,^ ^15^ ^,^ ^17^ ^,^ ^18^ ^,^ ^19^ ^,^ ^20^ ^,^ ^30^ ^,^ ^31^ ^,^ ^38^ ^,^ ^39^ ^,^ ^40^ ^,^ ^41^ ^,^ ^42^ ^,^ ^43^. This measurement method has been frequently employed, usually in population studies, using simple and direct questions that make it possible to aggregate the responses and construct variables that characterize the perceived disorder, allowing the assessment of constructs that cannot be measured by other methods ^39^, such as social disorder variables. However, individuals may respond differently based on their own behavior, thus resulting in common source bias. In other words, participants may be biased due to the stigma associated with low-income neighborhoods, being more likely to evaluate them with higher levels of disorder ^47^ ^,^ ^48^ ^,^ ^49^. It should also be considered that perception may be associated with individual characteristics, such as sex, age, and length of residence ^39^. The studies included in this review agree that the lack of objective measures of the environment is a limitation, as they may not always correlate with perceived measures ^17^ ^,^ ^18^ ^,^ ^19^ ^,^ ^20^ ^,^ ^39^ ^,^ ^41^.

The SSO, among the selected studies, was performed only in Brazil ^21^ ^,^ ^44^ ^,^ ^45^ ^,^ ^46^. This method allows recording, in a valid and reliable way, the physical attributes of the neighborhood, measuring characteristics that are not captured by census information, by other macro indicators, nor by the individuals’ perception ^50^. Moreover, it is a reproducible method that can work with other research techniques and survey quantitative and qualitative data in the same investigation. It also has the advantage of being the best option for areas that are difficult to access, where remote sensing or pre-collected images are not available ^45^ ^,^ ^46^. The way in which SSO can be applied can also vary, as was observed in the study by Remigio et al. ^46^, who developed a mobile app for systematic data collection in a large subnormal settlement in Rio de Janeiro. On the other hand, studies report that the use of SSO may have been limiting, as certain disorder items are subject to temporal variation. Thus, a more reliable measurement would require more than one observation, on different days and times, for the same street segment, as well as having more complex field logistics, which would result in high costs and extended data collection periods ^21^ ^,^ ^44^ ^,^ ^45^.

Recently, there has been a growth in the development and use of new methods to assess neighborhood attributes through emerging technologies ^49^. Among them is the virtual audit through Google Street View (https://www.google.com/maps), a digital alternative of SSO, which usually has a lower cost and less complex logistics. Some studies report that many variables of on-site audit can be assessed from remote imagery with reliability comparable to in-person assessment ^51^ ^,^ ^52^ ^,^ ^53^ ^,^ ^54^. Fry et al. ^55^ evaluated the availability of Google Street View images in 371 Latin American cities and observed that localities with better socioeconomic conditions tended to have more consistent images. It is worth mentioning that none of the articles selected in this review performed the virtual audit, which, in turn, has been used in previous studies related to the food environment ^56^ ^,^ ^57^ and physical activity ^58^.

The use of secondary data was also observed among the selected studies ^12^ ^,^ ^16^ ^,^ ^22^ ^,^ ^59^. Population census measures, for example, besides having many variables, cover several municipalities in countries that perform them, as was observed in Brazil and Colombia ^12^ ^,^ ^59^. However, they are collected only in certain periods and are not necessarily current. The geographic area is based on administrative units that may not represent social or geographic boundaries. Moreover, it usually contains limited variables about economic and structural factors, ignoring the social processes in the neighborhood ^47^ ^,^ ^48^. In the study by Auler et al. ^12^, conducted in three Brazilian capitals, the collection of neighborhood characteristics was carried out in person by the supervisors of the 2010 *Demographic Census*, representing a highlight in this set of information.

Among the selected studies, the geographic unit of analysis and data collection regarding disorder was mostly concentrated at the context level ^12^ ^,^ ^13^ ^,^ ^16^ ^,^ ^18^ ^,^ ^21^ ^,^ ^22^ ^,^ ^30^ ^,^ ^39^ ^,^ ^40^ ^,^ ^41^ ^,^ ^44^ ^,^ ^45^ ^,^ ^46^ ^,^ ^59^, mainly in neighborhoods ^13^ ^,^ ^18^ ^,^ ^22^ ^,^ ^30^ ^,^ ^39^ ^,^ ^40^. This geographical unit is a territory that can be subjectively or objectively delimited, where people live and interact socially, and is a measure of the macro-scale of the environment resulting from the aggregation of individual data or a smaller scale, which may reflect the characteristics of the context. For the assessment of larger areas, geographic information system-based measures are employed and consist of the set of tools for obtaining, storing, analyzing, and representing spatial data ^5^ ^,^ ^38^. On the other hand, the micro-scale is differentiated by describing the urban configuration, in terms of presence and quality of infrastructure, such as items measured at the street level (e.g., sidewalks and trees). Individuals’ perception and SSO constitute adequate methods for assessing smaller areas, and information obtained at the micro-scale can contribute to interventions with greater potential for change and lower costs ^45^ ^,^ ^58^.

From this scoping review, it was also possible to establish recommendations for future research on neighborhood disorder. For reviews using a systematic process, we recommend the use of automated tools, such as text mining, which enables automatic extraction of concepts and keywords, allowing reviews to be completed more quickly, as well as minimizing the impact of publication bias and reducing the chances of losing relevant research (recommendation 1) ^60^. Standardization of variables that compose the construct is also needed, since physical disorder relates to characteristics of the context (e.g., environmental factors, aesthetics, real estate and public facilities, and public streets) and social disorder relates to aspects of interaction between people and the context (e.g., neighborhood problems and security) (recommendations 2-4). It should be noted that methods that use objective measures are better suited to assess physical disorder, while those that use subjective measures are better suited to assess social disorder. As mentioned earlier, no studies were selected that used virtual auditing. Thus, we suggest the use of new methods to measure disorder, such as virtual audits via Google Street View, which is an efficient alternative to on-site audits and is safer for the auditors, performed in less time and with less financial resources. Moreover, it allows covering more study sites, such as large or distant areas, as well as acquiring historical images for longitudinal studies and application in computer vision models (recommendation 5) ^55^ ^,^ ^61^ (Box 5).

**Box 5:** Summary of recommendations for future research.

METHODOLOGICAL ISSUES		RECOMMENDATIONS FOR FUTURE RESEARCH	INTENDED IMPROVEMENTS
1	Systematic literature review	Use of automated tools, such as text mining	Automatic extraction of concepts and keywords; reviews completed more quickly; to minimize the impact of publication bias; to reduce the chances of losing relevant research; to assess the quality of studies; to produce more timely and reliable reviews
2	Concept of disorder	To standardize the concept of neighborhood disorder, taking into account the observed and perceived physical and social characteristics that may signal a disruption of order and social control	To compare results between studies; to synthesize the evidence; greater understanding
3	Physical disorder variables	To standardize the variables that constitute the construct of physical disorder of the neighborhood, considering that physical disorder is related to the characteristics of the context: variables related to water, air, soil and noise, aesthetics, real estate and public facilities, and public streets	To compare results between studies; to synthesize the evidence; greater understanding
4	Social disorder variables	To standardize the variables that constitute the social disorder construct of the neighborhood, considering that social disorder relates to aspects of interaction between people and the context: variables referring to neighborhood problems and security	To compare results between studies; to synthesize the evidence; greater understanding
5	New methods for measuring disorder	Use of virtual audits, such as Google Street View	Efficient alternative to on-site audits; to assess physical disorder; safe for auditors; shorter time; less financial resources; more study sites (large or distant areas); historical images for longitudinal studies; computer vision models

The results obtained through this review made it possible to observe the advancement in studies on environmental disorder. However, there is still no consensus on the items that measure physical and social neighborhood disorder in Latin American cities, which indicates the need for method standardization and future studies that evaluate the psychometric properties of the disorder constructs, as well as greater sophistication in the analytical approaches used. We consider as fundamental systematic review studies, meta-analysis and new evaluative studies that verify the continuity, systematization and implementation of new methods of measurement and analysis in urban health to assess neighborhood disorder in a continuous and longitudinal way in Latin American countries, since environmental disorder is an important construct for understanding the relationships between physical and socioeconomic neighborhood conditions and health outcomes.

This review had the limitation of using only the scientific literature, not including the gray literature. Also, the search strategy did not address the different terms used to describe disorder, such as neighborhood disturbances and problems, nor the terms used to describe methods of measuring disorder, which merits consideration in future work. The strengths of the study include the use of PRISMA-ScR guidelines to ensure a robust and replicable process and originality, as to our knowledge it is the first review on this theme in the Latin American context.

## Conclusion

This review revealed that the most commonly used method to measure neighborhood disorder in Latin America is the perception of the urban environment. Most studies examined adults and assessed both disorders, generally with composite indicators using scales. Moreover, the item most evaluated for physical disorder was related to the characteristics of public streets, while for social disorder, it was those related to security. The need to standardize the variables used to measure disorder, considering physical and social peculiarities separately can be seen from the findings. Furthermore, mixed methods of measurement are relevant to broaden the understanding of the phenomenon. Combining perception, systematic observation, and other methods will allow for capturing urban aspects that affect citizens’ health more accurately in future studies.
